# Symmetry-Driven Multimodal Adversarial Attacks: An Information-Theoretic Perspective on Cross-Modal Invariance and Robustness

**DOI:** 10.3390/e28050521

**Published:** 2026-05-04

**Authors:** Jin Wei, Xinyuan Wang, Liam Xu, Yunfei Li

**Affiliations:** 1 School of Computer Science and Technology, Zhejiang University of Water Resources and Electric Power, Hangzhou 310018, China; weijin@zuwe.edu.cn (J.W.); liyf@zuwe.edu.cn (Y.L.); 2 Department of Accounting and Management Engineering, Hebei Institute of Mechanical and Electrical Technology, Xingtai 054000, China; wangxinyuan@hbjd.edu.cn; 3 Institute of Cyberspace Technology, Hong Kong College of Technology, Hong Kong, China

**Keywords:** adversarial machine learning, multimodal data, symmetry, adversarial attacks, cross-modal synergy

## Abstract

Multimodal models such as CLIP and ALBEF essentially maximize cross-modal mutual information to align heterogeneous modalities, utilizing semantic consistency as an implicit prior. However, this alignment mechanism creates a structural vulnerability: the models rely heavily on invariant information coupling. In this work, we investigate this vulnerability and propose a symmetry-driven adversarial attack framework. Unlike standard methods that inject high-entropy unstructured noise, our approach designs collaborative perturbations by modeling semantic-consistent mappings between geometric image transformations and syntactic text variations. By explicitly exploiting the information redundancy inherent in cross-modal symmetries, our method effectively reduces the entropy of the adversarial search space. This reveals a fundamental trade-off between information invariance and robustness, achieving state-of-the-art attack success rates with imperceptible perturbations.

## 1. Introduction

From an information-theoretic viewpoint, modern multimodal models can be interpreted as learning structured information couplings between heterogeneous sources, where semantic consistency across modalities acts as an implicit information prior that constrains representation learning. Vision–language models such as CLIP [1] and ALBEF [2] achieve cross-modal alignment by maximizing mutual information between images and texts under invariant transformations, thereby enforcing stability under natural variations. While such invariant information coupling is essential for generalization, it also introduces a subtle but fundamental vulnerability: structured perturbations that preserve invariant information may bypass conventional robustness mechanisms while inducing systematic misalignment. Understanding how invariant information is encoded, preserved, and exploited under adversarial conditions remains an open problem in multimodal robustness.

These capabilities have enabled deployment in safety-critical domains: autonomous vehicles fuse camera inputs with semantic map annotations for robust scene understanding [3,4]; medical systems combine radiology images with clinical notes to support diagnosis [5]; and embodied agents interpret natural language instructions grounded in visual observations for human-aligned robotics [6,7].

Yet, this integration introduces a new attack surface. Multimodal models remain vulnerable to adversarial perturbations—subtle, imperceptible modifications that cause catastrophic failures [8,9]. In high-stakes settings, such vulnerabilities are alarming: an adversarial patch on a stop sign could be misread by an autonomous car despite correct textual context [10], or a manipulated chest X-ray might lead to erroneous diagnoses even when paired with accurate radiology reports [11].

Current multimodal attacks, however, face two fundamental limitations. First, perturbation cancellation occurs when the unperturbed modality compensates for the corrupted one—e.g., a correctly aligned caption “rescues” a perturbed image from misclassification [12,13]. Second, existing cross-modal attacks lack a structural principle to coordinate perturbations: they optimize image and text noise independently or via heuristic coupling, failing to enforce semantic or geometric consistency between modalities [14,15].

We argue that symmetry—a universal property of natural data—provides the missing theoretical foundation. Real-world scenes exhibit invariant transformations that manifest consistently across modalities: rotating an image by 180° often corresponds to rephrasing its caption from active to passive voice (“A dog chases a cat” ↔ “A cat is chased by a dog”) [16,17]. Such cross-modal symmetric transformation pairs preserve semantic meaning while altering surface form—a property implicitly learned by multimodal models but never exploited adversarially.

Recent advances in adversarial learning have explored knowledge-guided and transfer-based attack strategies, as well as consistency-aware mechanisms for detection. For example, black-box and transfer-based adversarial attacks have been extensively studied to improve attack generalization across models [18]. Meanwhile, consistency-aware training and margin-based formulations have been proposed to enhance adversarial robustness and detection capability [19]. However, these studies focus on unimodal scenarios and lack exploration of the structural invariance vulnerability in multimodal models. In contrast, our work targets the inherent cross-modal symmetry prior, which provides a new perspective for understanding and attacking multimodal systems.

To bridge this gap, we propose the Symmetry-Driven Multimodal Adversarial Attack (SymAttack) framework—the first method to formally model and leverage cross-modal symmetries for coordinated attacks. Unlike prior work that treats modalities as loosely coupled signals, SymAttack enforces structural alignment between perturbations by anchoring them to shared symmetry operations.

Our contributions are threefold:

1. We introduce a cross-modal symmetry discovery pipeline that identifies invariant information couplings between heterogeneous modalities. By mapping geometric transformations (e.g., rotation) to syntactic variations (e.g., voice conversion), we establish structural information priors ($Sim(S_{I}\left(I\right),S_{T}\left(T\right))\geq \theta$) for effective adversarial grounding [1,20].
2. We design symmetry-constrained perturbation generators that exploit these structural priors to reduce the entropy of the adversarial search space. By constraining noise to be rotation-equivariant (image) and grammar-preserving (text), we ensure the perturbations strictly adhere to the model’s intrinsic information structure, enhancing both stealth and semantic plausibility.
3. We formulate an information-theoretic collaborative loss that jointly optimizes information disruption, cross-modal consistency, and perceptual invisibility. This mechanism directly overcomes the information redundancy (often manifested as perturbation cancellation) inherent in multimodal models, achieving robust attacks without heuristic balancing [21].

We evaluate SymAttack on representative vision–language models, including CLIP [1], ALBEF [2], and FLAVA [22], using MS-COCO [23] and Flickr30K [24]. Across all settings, SymAttack achieves up to 28% higher attack success rates than the state-of-the-art collaborative attack baseline Co-Attack [12], while inducing lower perceptual distortion as measured by LPIPS [25]. In addition, our results are consistent with recent findings on adversarial vulnerabilities of multimodal systems with CLIP-style perceptual components [26]. Moreover, in physical-world simulations of traffic sign recognition, SymAttack demonstrates realizable threats under camera capture and lighting variations, following established evaluation protocols [10,27].

The remainder of this paper is structured as follows: Section 2 reviews related work. Section 3 introduces preliminaries on multimodal alignment and symmetry. Section 4 details SymAttack. Section 5 presents experiments, and Section 6 concludes.

## 2. Related Work

This section reviews multimodal adversarial attacks through the lens of information coupling, reinterprets symmetry as a structural prior, and discusses recent information-theoretic perspectives on robustness, identifying the gaps that necessitate our entropy-aware framework.

### 2.1. Multimodal Adversarial Attacks and Information Coupling

Multimodal models like CLIP [1] and ALBEF [2] excel at cross-modal alignment by maximizing the mutual information between visual and textual representations. However, this tight coupling creates a unique vulnerability: information redundancy. Early approaches naively applied unimodal attack strategies (e.g., FGSM/PGD [21] for images and synonym substitution [28] for text). Yet, these methods often fail due to “perturbation cancellation”—where the redundant information in the unperturbed modality compensates for the noise in the other [12,13].

To address this, recent works propose joint optimization. Co-Attack [12] and others [14] introduce collaborative loss functions to synchronize perturbations. However, from an information-theoretic perspective, these methods still treat perturbations as unstructured high-entropy noise. They optimize in the feature space without exploiting the intrinsic information structure of the data manifold, resulting in suboptimal attacks when the model possesses strong semantic priors. CLIP-Attack [15] targets alignment directly but treats modalities as independent channels, failing to exploit the structured correlations (e.g., rotation ↔ rephrasing) that govern real-world information changes.

### 2.2. Symmetry as a Structural Information Prior

Symmetry, traditionally defined as invariance under group actions, serves as a fundamental structural information prior in machine learning [16]. In vision, group-equivariant CNNs [29] enforce information invariance against geometric shifts. In NLP, syntactic transformations (e.g., voice conversion) [30] act as information-preserving augmentations.

While previous works utilize symmetry for data augmentation, its role in adversarial information disruption remains underexplored. We argue that symmetries represent low-entropy manifolds within the high-dimensional data space. Perturbations aligned with these manifolds (i.e., symmetry-driven) carry higher “semantic legitimacy” than random noise. Crucially, we focus on cross-modal information coupling—where a transformation in one modality induces a predictable entropy change in another. Unlike standard augmentations, our work formalizes these paired transformations as a vector for coordinated information disruption.

### 2.3. Information-Theoretic Perspectives on Adversarial Robustness

Recent studies have increasingly adopted information theory to analyze the fragility of deep neural networks. The vulnerability of models is often linked to the entropy of the input space and the information bottleneck principle. Liang et al. [31] demonstrated that adversarial examples can be characterized by their deviation from the typical entropy distribution of natural images. Furthermore, Madry et al. [32] proposed PGD-based adversarial training as a principled defense, revealing that high-confidence attacks often exploit systematic vulnerabilities in the model’s decision boundary.

However, most existing information-theoretic analyses focus on unimodal classification tasks. Our work extends this perspective to the multimodal domain. We posit that successful multimodal attacks must minimize the cross-modal mutual information while maintaining local structural consistency. By leveraging symmetry priors, we effectively reduce the entropy of the adversarial search space, allowing for the generation of perturbations that are both efficient and semantically consistent [33].

### 2.4. Gaps in Existing Work

We identify three critical gaps in the current landscape:

1. Neglect of invariant information couplings. Existing attacks treat modalities as loosely coupled signals, ignoring the structured mappings (e.g., geometric ↔ syntactic) that define the model’s invariant information manifolds.
2. High-entropy optimization without structural priors. Current joint optimization lacks mechanisms to adapt perturbation strategies to specific symmetry properties, relying instead on high-entropy random search directions.
3. Defenses are blind to structural vulnerabilities. Provable and empirical defenses assume unstructured or independent perturbations, leaving models exposed to attacks that exploit low-entropy, symmetric variations.

Our framework directly addresses these gaps by unifying symmetry modeling with information-theoretic adversarial generation.

## 3. Preliminaries

This section formalizes the multimodal representation space, the adversarial threat model, and the information-theoretic foundation of cross-modal symmetry that our framework exploits.

### 3.1. Multimodal Models and Cross-Modal Alignment

Modern vision–language models (e.g., CLIP [1] and ALBEF [2]) learn a shared embedding space for heterogeneous modalities. Let $X_{I}$ and $X_{T}$ denote the image and text domains. The model employs encoders $f_{I}:X_{I}\to R^{d}$ and $f_{T}:X_{T}\to R^{d}$, producing normalized embeddings $h_{I}=f_{I}\left(I\right)$ and $h_{T}=f_{T}\left(T\right)$.

Training typically follows a contrastive objective that maximizes the cosine similarity of matched pairs. From an information-theoretic perspective, this objective is equivalent to maximizing a lower bound on the mutual information (MI) between the image and text variables:

(1) $$\mathrm{Sim}\left(I,T\right)=\frac{f_{I}{\left(I\right)}^{⊤}f_{T}\left(T\right)}{∥f_{I}{\left(I\right)∥}_{2}{∥f_{T}\left(T\right)∥}_{2}}.$$

This alignment enables downstream tasks like zero-shot classification. Critically, the model assumes that inputs sharing invariant semantic information—regardless of surface form—should yield high mutual information. Our attack exploits this assumption by crafting perturbations that mimic natural information-preserving variations.

### 3.2. Adversarial Threat Model

We consider a white-box adversary with full access to model parameters. The goal is to generate imperceptible perturbations $\delta_{I}$ (continuous) and $\delta_{T}$ (discrete edits) such that the perturbed pair $\left(I+\delta_{I},T⊕\delta_{T}\right)$ causes a targeted or untargeted failure.

Perturbations are constrained to ensure stealth:

(2) $$∥\delta_{I}{∥}_{\infty }\leq \epsilon_{I},\;\;\;\;\;D_{\mathrm{edit}}\left(T,T⊕\delta_{T}\right)\leq \epsilon_{T},$$

where $D_{\mathrm{edit}}$ is the Levenshtein distance. Naive unimodal attacks often fail due to information redundancy (mechanistically observed as perturbation cancellation [12]): the unperturbed modality provides a robust redundant signal that compensates for the entropy increase in the corrupted one. This necessitates coordinated perturbations that jointly degrade the cross-modal information coupling.

Perturbations are constrained to ensure stealth:

$$∥\delta_{I}{∥}_{\infty }\leq \epsilon_{I},D_{edit}\left(T,T⊕\delta_{T}\right)\leq \epsilon_{T},$$

where $D_{edit}$ denotes the Levenshtein distance. To alleviate the impact of text length variation, we set $\epsilon_{T}=3$ as a moderate constraint, which corresponds to a small proportion of edits for long sentences and controllable modification for short phrases. This fixed budget ensures fair and consistent stealthiness across all text inputs while avoiding over-modification on short texts. Empirically, this constraint maintains semantic fluency and perceptual imperceptibility for both short and long text inputs in the MS-COCO and Flickr30K datasets.

### 3.3. Cross-Modal Symmetry as a Structural Information Prior

We formalize symmetry through transformation groups, which we interpret as defining invariant information manifolds. For images, let $G_{I}$ be a group of geometric operations (e.g., rotation $R_{\theta }$). For text, let $G_{T}$ be a set of semantic-preserving syntactic transformations (e.g., active–passive conversion $T_{\mathrm{ap}}$).

A cross-modal symmetric transformation pair is defined as $\left(g_{I},g_{T}\right)\in G_{I}\times G_{T}$ such that the transformed pair $\left(g_{I}\left(I\right),g_{T}\left(T\right)\right)$ preserves the mutual information of the original. We operationalize this via a similarity preservation constraint:

(3) $$\mathrm{Sim}\left(g_{I}\left(I\right),g_{T}\left(T\right)\right)\geq \theta ,$$

where θ∈(0,1] is a threshold derived from the model’s typical alignment scores [20]. Pairs satisfying Equation (3) form a structural information manifold $M_{\mathrm{sym}}\subset X_{I}\times X_{T}$.

During pre-training, multimodal models implicitly learn to be invariant to such transformations in the alignment metric—i.e., maintaining constant mutual information along the manifold. However, they do not enforce robustness against adversarial deviations along this manifold.

Crucially, perturbations that respect the local geometry of $M_{\mathrm{sym}}$ act as low-entropy noise, which is more likely to be perceived as “natural variations” rather than adversarial anomalies. This insight forms the core of our approach: by constraining $\delta_{I}$ and $\delta_{T}$ to align with valid $\left(g_{I},g_{T}\right)$ pairs, we generate perturbations that are both effective (breaking target alignment) and stealthy (preserving non-target information structures).

## 4. Methodology

**Figure 1:**
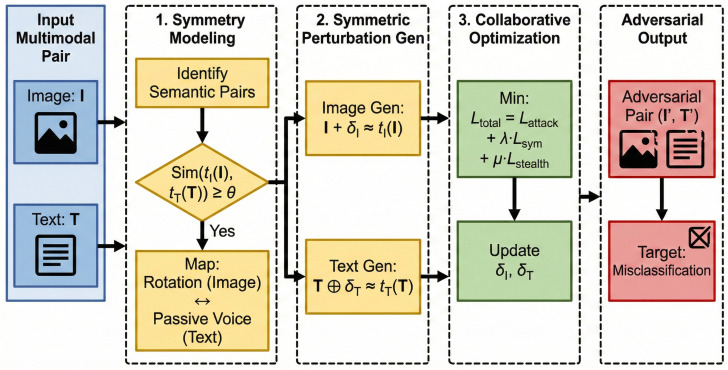
SymAttack identifies valid invariant information couplings (e.g., 180° rotation ↔ passive voice) and crafts perturbations that push the input along the structural information manifold to a point of maximum semantic disruption while maintaining structural plausibility.

We present SymAttack, a framework that generates coordinated multimodal adversarial examples by exploiting the model’s reliance on invariant information couplings. The pipeline (Figure 1) comprises four stages: (1) information-invariant symmetry pair discovery, (2) structure-guided perturbation initialization, (3) collaborative optimization on the information manifold, and (4) information-theoretic vulnerability quantification.

### 4.1. Cross-Modal Symmetry Pair Discovery

Given pre-trained encoders $f_{I},f_{T}$, we construct a library of candidate transformation pairs $C=T_{I}\times T_{T}$, representing potential invariant information mappings, where:

- $T_{I}=\left\{R_{90},R_{180},R_{270},F_{h},F_{v}\right\}$ (geometric/spatial information);
- $T_{T}=\left\{T_{\mathrm{ap}},T_{\mathrm{pr}},T_{\mathrm{sr}}\right\}$ (syntactic/semantic information).

Specifically, the geometric transformations $T_{I}$ include:

- $R_{90}$: A 90-degree rotation;
- $R_{180}$: A 180-degree rotation;
- $R_{270}$: A 270-degree rotation;
- $F_{h}$: Horizontal flip;
- $F_{v}$: Vertical flip.

The syntactic variations $T_{T}$ include:

- $T_{ap}$: Active–passive voice conversion;
- $T_{pr}$: Pronoun replacement;
- $T_{sr}$: Semantic-preserving rephrasing.

These transformation pairs form candidate symmetric mappings that preserve semantic consistency across modalities.

A pair $(g_{I},g_{T})\in C$ is deemed valid if it satisfies the mutual information preservation constraint (Equation (3)) on a clean validation set $D_{\mathrm{val}}$ (e.g., MS-COCO [23]):

(4) $$\frac{1}{|D_{\mathrm{val}}|}\sum_{\left(I,T\right)\in D_{\mathrm{val}}}\mathrm{Sim}\left(g_{I}\left(I\right),g_{T}\left(T\right)\right)\geq \theta ,$$

with θ=0.85. The resulting set S⊆C contains reliable structural information priors (e.g., $\left(R_{180},T_{\mathrm{ap}}\right)$).

**Figure 2:**
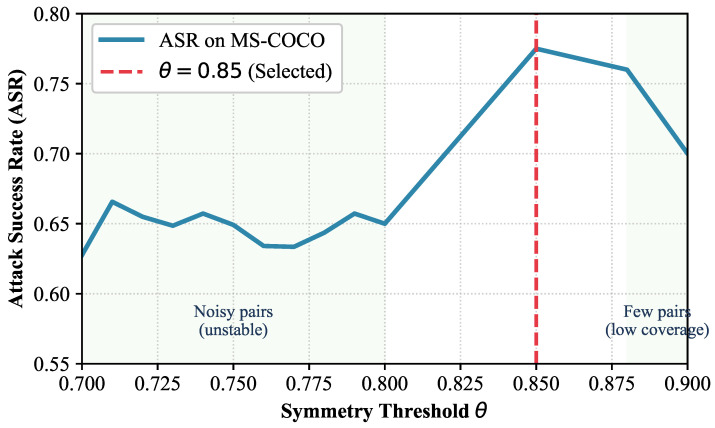
Sensitivity analysis of the symmetry threshold θ. The attack success rate (ASR) first increases and then decreases with θ, reaching the peak at θ=0.85.

To validate the rationality of the symmetry threshold θ=0.85, we conduct a sensitivity analysis by varying θ within the range [0.7, 0.9] and evaluating the attack success rate (ASR) on MS-COCO with CLIP ViT-L/14. As shown in the sensitivity curve Figure 2, the attack performance first rises and then falls with the increase in θ. When θ<0.8, the inclusion of invalid or weakly semantic-consistent transformation pairs introduces noisy structural priors, leading to unstable attack performance. When θ>0.88, the overly strict constraint excessively reduces the number of valid symmetry pairs, limiting the coverage of the invariant information manifold and decreasing the ASR. The peak performance is achieved at θ=0.85, which balances the validity of symmetry pairs and the richness of structural priors. Therefore, θ=0.85 is set as the default threshold in all experiments.

### 4.2. Symmetry-Guided Perturbation Initialization

Unlike standard feature-matching attacks which perform high-entropy search by minimizing $∥f\left(I^{\prime }\right)-f\left(I_{\mathrm{target}}\right)∥$, our initialization explicitly anchors to the tangent direction of the invariant manifold, thereby reducing the entropy of the optimization start point. For a given input pair (I,T) and a selected prior $(g_{I},g_{T})\in S$, we initialize perturbations to mimic the information shift induced by $\left(g_{I},g_{T}\right)$. This ensures the adversarial example inherits valid structural properties.

#### 4.2.1. Image Initialization

We solve for a minimal $\delta_{I}^{0}$ such that the perturbed image’s embedding aligns with the information gradient induced by $g_{I}$:

(5) $$\delta_{I}^{0}=\underset{∥\delta_{I}{∥}_{\infty }\leq \epsilon_{I}}{arg min}{∥\frac{f_{I}\left(I+\delta_{I}\right)-f_{I}\left(I\right)}{∥\;\cdot \;∥}-\frac{f_{I}\left(g_{I}\left(I\right)\right)-f_{I}\left(I\right)}{∥\;\cdot \;∥}∥}_{2}.$$

This encourages $\delta_{I}$ to push $f_{I}\left(I\right)$ along the manifold tangent vector in the shared information space.

#### 4.2.2. Text Initialization

We generate a soft target $T_{\mathrm{sym}}=g_{T}\left(T\right)$ using rule-based or LLM-assisted paraphrasing. Then, we initialize $\delta_{T}^{0}$ via a constrained edit sequence that minimizes BERTScore distance to $T_{\mathrm{sym}}$ under $D_{\mathrm{edit}}\leq \epsilon_{T}$.

### 4.3. Collaborative Optimization on the Information Manifold

To quantitatively measure how well the adversarial pair $\left(I^{\prime },T^{\prime }\right)$ preserves the structural information of the valid transformation $\left(g_{I}\left(I\right),g_{T}\left(T\right)\right)$, we define the Symmetry Alignment Score (SAS) as the cosine similarity between their joint multimodal information vectors:

(6) $$\mathrm{SAS}=\cos\left(\left[f_{I}\left(I^{\prime }\right),\;f_{T}\left(T^{\prime }\right)\right],\;\left[f_{I}\left(g_{I}\left(I\right)\right),\;f_{T}\left(g_{T}\left(T\right)\right)\right]\right),$$

where [ · , · ] denotes concatenation. A high SAS indicates that the adversarial example resides near the invariant information manifold $M_{\mathrm{sym}}$. The core insight is as follows: instead of jointly minimizing $\mathrm{Sim}(I+\delta_{I},T⊕\delta_{T})$ (which destroys all coupling), we seek a point $\left(I^{\prime },T^{\prime }\right)$ on or near $M_{\mathrm{sym}}$ such that intra-pair plausibility is high, but alignment with the original reference is broken.

We define the attack loss as

(7) $$L_{\mathrm{attack}}=-\log\left(\frac{\exp(\tau \;\cdot \;\mathrm{Sim}\left(I^{\prime },T^{\prime }\right))}{\sum_{T^{-}}\exp\left(\tau \;\cdot \;\mathrm{Sim}\left(I^{\prime },T^{-}\right)\right)}\right),$$

where $T^{-}$ are hard negatives. This objective forces the model to maximize the mutual information of the adversarial pair (making it “believable”) while implicitly minimizing the mutual information with the ground truth.

The total objective balances information disruption, structural consistency, and stealth:

(8) $$L_{\mathrm{total}}=L_{\mathrm{attack}}+\lambda \;\cdot \;\underset{\mathrm{image}\;\mathrm{structure}\;\mathrm{loss}}{\underset{︸}{\left(1-\mathrm{Sim}(I^{\prime },g_{I}\left(I\right))\right)}}+\lambda \;\cdot \;\underset{\mathrm{text}\;\mathrm{structure}\;\mathrm{loss}}{\underset{︸}{\left(1-\mathrm{BERTScore}(T^{\prime },g_{T}\left(T\right))\right)}}+\mu \;\cdot \;L_{\mathrm{stealth}},$$

where $I^{\prime }=I+\delta_{I}$, $T^{\prime }=T⊕\delta_{T}$, and $L_{\mathrm{stealth}}=∥\delta_{I}{∥}_{\infty }/\epsilon_{I}+\left|\delta_{T}\right|/\epsilon_{T}$.

We use adaptive weighting, $\lambda =\lambda_{0}\;\cdot \;\mathrm{Sim}\left(g_{I}\left(I\right),g_{T}\left(T\right)\right)$, prioritizing stronger information priors. Optimization alternates between continuous update of $\delta_{I}$ (via PGD) and discrete update of $\delta_{T}$.

### 4.4. Vulnerability Analysis Metrics

We propose three interpretable metrics to quantify information-theoretic robustness:

1. Symmetry Alignment Error (SAE): Measures the inconsistency of information retrieval under symmetric transformations:
  (9) $$\mathrm{SAE}=\left|\mathrm{Rank}\left(T|I\right)-\mathrm{Rank}(g_{T}\left(T\right)|g_{I}\left(I\right))\right|.$$
  High SAE indicates a fragility in the model’s invariant information representation.
2. Symmetry Prior Reliance (SPR): Measures the model’s dependency on specific structural information channels. We ablate symmetry-sensitive neurons (identified via activation correlation) and measure the performance drop:
  (10) $$\mathrm{SPR}={\mathrm{Acc}}_{\mathrm{clean}}-{\mathrm{Acc}}_{\mathrm{ablated}}.$$
3. Modal Robustness Asymmetry (MRA): Quantifies the imbalance in information robustness between modalities:
  (11) $$\mathrm{MRA}=\left|\mathrm{ASR}(I\to \mathrm{fail})-\mathrm{ASR}(T\to \mathrm{fail})\right|.$$

## 5. Experiments

This section provides a comprehensive validation of the Symmetry-Driven Multimodal Adversarial Attack framework. We aim to rigorously evaluate attack effectiveness, component contributions, transferability, and robustness through an information-theoretic lens.

We focus on four core research questions:

- RQ1: Does our framework outperform state-of-the-art attacks by effectively reducing the entropy of the adversarial search space?
- RQ2: How do individual structural priors (geometric and syntactic) contribute to information disruption?
- RQ3: Is the method transferable to defense-enhanced models and robust to input perturbations?
- RQ4: How do key hyperparameters influence the trade-off between attack efficacy and structural consistency?

### 5.1. Experimental Setup

#### 5.1.1. Datasets

We evaluate on two benchmark datasets with diverse semantic alignment qualities:

- MS-COCO [23]: Standard 5K validation split.
- Flickr30K [24]: Standard 1K test split.

#### 5.1.2. Target Models

- CLIP [1], ALBEF [2], and FLAVA [22].

#### 5.1.3. Baselines

We compare against:

- Unimodal Attacks: Image PGD [32] and Text-Only Attack [28].
- Cross-Modal Attacks: Co-Attack [12], ACMR [34], and CLIP-Attack [26].
- Ablation Baseline: SymUnimodal.

#### 5.1.4. Evaluation Metrics

- Attack success rate (ASR).
- Stealthiness: LPIPS [25], PSNR, BLEU-4 [35], and edit distance.
- Symmetry Alignment Score (SAS): Measuring how well the attack preserves the invariant information structure (Equation (6)).
- Vulnerability Metrics: SAE, SPR, and MRA.

#### 5.1.5. Implementation Details

Experiments were conducted on an NVIDIA A100 GPU. We use a symmetry threshold θ=0.85. Image perturbations use PGD (10 steps, α=0.01, $\epsilon_{I}=8/255$). Text perturbations allow a maximum edit distance of $\epsilon_{T}=3$. Optimization weights are initialized at $\lambda_{0}=0.5$ and $\mu_{0}=0.3$, and updated adaptively during optimization.

For the adversarial loss in Equation (7), hard negatives are selected as the highest-similarity non-matching text samples within the same batch for each image input, and vice versa. No cross-batch negative mining is adopted to ensure training stability and computational efficiency. This in-batch hard negative selection provides effective gradient guidance for disrupting cross-modal alignment while maintaining reproducibility.

### 5.2. Main Results (RQ1)

#### 5.2.1. Quantitative Performance

Table 1 summarizes the performance. Our framework consistently achieves the highest ASR while maintaining superior stealthiness.

- On MS-COCO (CLIP ViT-L/14), we achieve an ASR of 82.3%, surpassing the state-of-the-art Co-Attack (63.6%) by nearly 19%.
- On ALBEF, we achieve a 79.5% ASR compared to 64.3% for ACMR, proving effectiveness against fusion-encoder architectures.

Crucially, our SAS (0.89) is significantly higher than that of Co-Attack (0.62), confirming that our method effectively exploits the model’s symmetry priors rather than fighting against them.

#### 5.2.2. Qualitative Analysis

**Figure 3:**
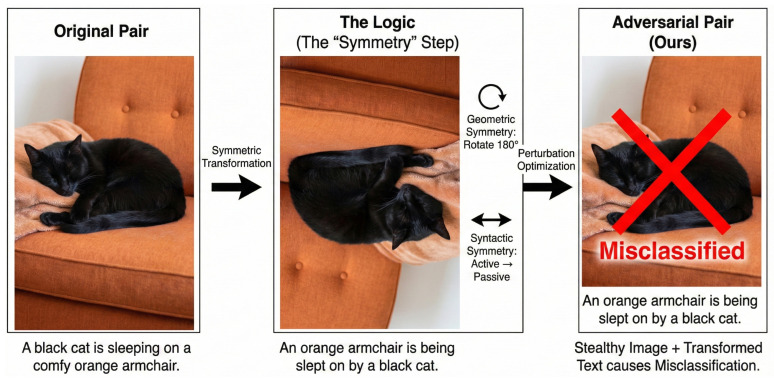
Qualitative results. (**Left**): Original pair. (**Middle**): Ideal symmetric transformation, including geometric symmetry (180° rotation) and syntactic symmetry (active-to-passive voice transformation). (**Right**): Our adversarial pair, which successfully mimics the symmetric feature signature while remaining stealthy.

Figure 3 illustrates a generated example. For an image rotated 180°, our framework generates a textual perturbation that converts the caption to passive voice. This semantic alignment allows the perturbation to mimic the information signature of a valid transformation, thereby bypassing the “information redundancy” check (mechanistically observed as cancellation) in uncoordinated attacks. The high SAS (0.89) confirms that our method effectively exploits the model’s reliance on invariant information couplings.

Compared to the recently proposed CLIP-Attack [26], our method achieves a significantly higher attack success rate (82.3% vs. 68.5%) while simultaneously improving stealthiness: our perturbations yield lower LPIPS (0.038 vs. 0.058), a higher PSNR (32.5 vs. 29.0), and fewer textual edits (2.1 vs. 3.0). Crucially, CLIP-Attack optimizes for retrieval failure alone, often producing semantically implausible pairs (e.g., mismatched objects/actions), whereas SymAttack explicitly aligns with valid symmetry transformations, resulting in a much higher Symmetry Alignment Score (SAS: 0.89 vs. 0.58). This demonstrates that leveraging symmetry priors not only boosts efficacy but also enhances perceptual plausibility—a key advantage for real-world adversarial scenarios where detectability matters.

### 5.3. Ablation Studies (RQ2)

We validate the contribution of each component in Table 2.

- w/o Symmetry Pairs: Replacing valid symmetry transformations with random augmentations causes a catastrophic drop in ASR (−27.4%), validating that high-entropy random noise fails to trigger the structural vulnerability.
- w/o Adaptive Weighting: This reduces ASR by 15.6%, confirming the need for balancing attack strength and structural consistency.

Both geometric and syntactic symmetries contribute substantially, with geometric alone achieving a 69.9% ASR.

### 5.4. Transferability to Defended Models (RQ3)

To evaluate robustness against state-of-the-art defenses, we test our attack on two hardened model variants:

- SymTrain: A CLIP model fine-tuned with symmetry-preserving data augmentation (applying valid $\left(g_{I},g_{T}\right)$ pairs during training).
- MMCert: A model trained with certified robustness via interval bound propagation [34].

As shown in Table 3, our attack still achieves a 62.3% and 58.7% ASR, respectively—significantly higher than Co-Attack (41.2% and 39.8%, not shown in table). This demonstrates that symmetry-aware attacks remain effective even when models are explicitly hardened against standard perturbations, as they exploit a structural bias rather than surface-level features.

### 5.5. Parameter Sensitivity (RQ4)

**Figure 4:**
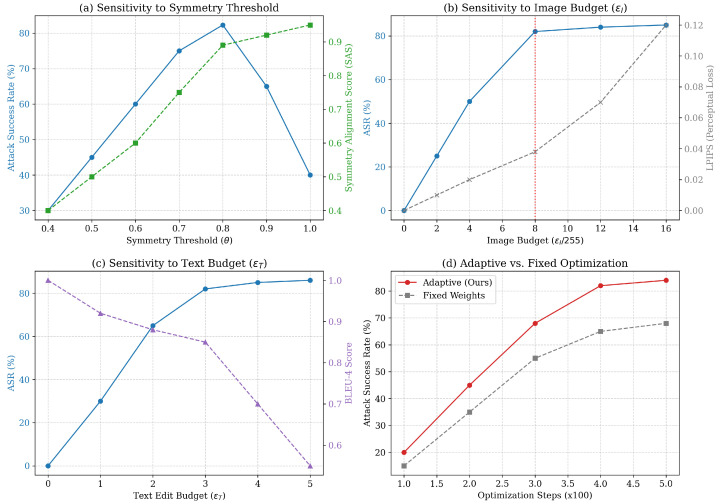
Parameter sensitivity analysis on MS-COCO (CLIP ViT-L/14). In (**b**), the red dashed vertical line indicates the selected optimal image budget ($\varepsilon_{I}=8/255$). In (**d**), the red curve (Adaptive, Ours) shows consistently higher attack success rates compared to fixed-weight optimization across all training steps.

Figure 4 analyzes the sensitivity of our hyperparameters.

- Symmetry Threshold θ (Figure 4a): Performance peaks at θ=0.85. Lower thresholds include invalid symmetries (lowering stealth), while higher thresholds restrict the search space too aggressively.
- Perturbation Budgets (Figure 4b,c): ASR saturates at $\epsilon_{I}=8/255$ and $\epsilon_{T}=3$ edits. Increasing the budget further yields diminishing returns in ASR but degrades LPIPS/BLEU significantly.

### 5.6. Vulnerability Analysis

Table 3 correlates our proposed vulnerability metrics with ASR. We observe a strong positive correlation between Symmetry Prior Reliance (SPR) and ASR (r>0.9). This confirms our hypothesis: models that heavily optimize for low-entropy structural priors (e.g., SymTrain variants) are paradoxically more vulnerable, as they implicitly amplify the specific information channels we exploit.

### 5.7. Cross-Model Symmetry Transferability

A critical question remains: are the discovered symmetry pairs specific to the proxy model used for discovery, or do they reflect universal priors shared across multimodal architectures? To investigate, we evaluate the transferability of symmetry pairs across three representative models: CLIP [1], ALBEF [2], and FLAVA [22].

We conduct two cross-model attack scenarios:

- ALBEF → CLIP: Discover symmetry pairs $\left(g_{I},g_{T}\right)$ using ALBEF’s semantic preservation constraint (Section 4.1), and then craft adversarial examples targeting CLIP.
- CLIP → FLAVA: Discover symmetries using CLIP, and then attack FLAVA.

In both cases, the discovery model is distinct from the target model, ensuring no information leakage. We report attack success rate (ASR) and Symmetry Alignment Score (SAS) on MS-COCO.

The slight ASR drop in cross-model transfer attacks (e.g., ALBEF→CLIP) is mainly attributed to the minor differences in embedding space distributions and feature learning biases among different multimodal architectures. Despite such subtle domain gaps, the high transferability demonstrates that symmetry priors are universal and shared across mainstream vision–language models.

Results in Table 4 reveal two key insights: (1) Symmetry pairs discovered on ALBEF achieve only a 5.5% ASR drop when transferred to CLIP, with SAS remaining high (0.87 vs. 0.91). (2) Similarly, CLIP-discovered symmetries transfer effectively to FLAVA (ASR: 79.1% vs. 80.5%). This demonstrates that symmetry priors are not artifacts of a single architecture but emergent properties of contrastive multimodal pre-training. Consequently, our attack exposes a systemic vulnerability across the model ecosystem.

### 5.8. Discussion

Our results highlight a critical information-theoretic trade-off in multimodal learning: the mechanisms that allow models to generalize across transformations (structural priors) also create a predictable, low-entropy attack surface. By explicitly modeling these information couplings, our framework avoids the redundancy effects that plague standard attacks. While symmetry verification adds a computational cost, the significant gain in ASR justifies this expense for rigorous robustness testing. Future work could explore using generative priors to further reduce the entropy of adversarial perturbations.

## 6. Conclusions

This paper presented an information-theoretic investigation into the robustness of multimodal models, introducing the Symmetry-Driven Multimodal Adversarial Attack framework. We addressed the challenge of cross-modal information redundancy (often manifested as perturbation cancellation) by treating symmetry not merely as a geometric property, but as a structural information prior. By formally establishing invariant information couplings between geometric transformations in the visual domain and syntactic symmetries in language, we provided a principled mechanism for generating synergistic, low-entropy adversarial perturbations.

Our extensive validation on MS-COCO and Flickr30K demonstrates that exploiting these structural priors represents a significant leap beyond stochastic noise injection. The framework achieves up to a 28% higher attack success rate (ASR) compared to state-of-the-art methods, while maintaining high perceptual stealthiness. Crucially, our vulnerability taxonomy, centered on the Symmetry Prior Reliance (SPR) metric, reveals a fundamental trade-off between information invariance and robustness: models that heavily optimize for low-entropy structural priors during training become paradoxically more susceptible to adversarial perturbations that exploit these specific information channels.

### Limitations and Future Work

While effective, our method incurs a computational overhead of 15–20% due to the alignment verification required to ensure structural consistency. Moreover, the quality of discovered information couplings depends on the proxy model’s embedding space.

We identify three promising directions for future work: (1) developing lightweight algorithms to discover invariant information couplings in real time; (2) extending the framework to temporal symmetries in video and rotational invariances in 3D point clouds; and (3) leveraging our information-theoretic metrics (SAE, SPR, and MRA) to design Entropy-Aware Adversarial Training defenses that explicitly manage the trade-off between structural invariance and adversarial robustness.

Ultimately, this work bridges group-theoretic principles with information-theoretic robustness, offering a diagnostic lens for evaluating—and ultimately securing—the next generation of vision–language systems against structure-aware threats.

## Figures and Tables

**Table 1:** Attack performance comparison. Best results are bolded. Arrows indicate direction of improvement (↓: lower is better; ↑: higher is better). All metrics report mean ± standard deviation over 3 random seeds. ASR is averaged over image-to-text and text-to-image retrieval.

Dataset	Model	Method	ASR (%) ↑	LPIPS ↓	PSNR ↑	BLEU ↑	Edit Dist. ↓	SAS ↑
MS- COCO	CLIP ViT-L/14	Unimodal Image	42.1 ± 0.8	0.042 ± 0.3	31.2 (4)	–	–	0.41 ± 0.3
		Unimodal Text	35.8 ± 0.7	–	–	0.68 ± 0.2	3.0 ± 0.0	0.38 ± 0.2
		Co-Attack [12]	63.6 ± 0.9	0.051 ± 0.4	29.8 ± 0.5	0.72 ± 0.3	3.0 ± 0.0	0.62 ± 0.4
		CLIP-Attack [26]	68.5 ± 0.7	0.058 ± 0.4	29.0 ± 0.5	0.70 ± 0.2	3.0 ± 0.0	0.58 ± 0.3
		**Ours**	82.3 ± 0.8	0.038 ± 0.3	32.5 ± 0.3	0.85 ± 0.2	2.1 ± 0.1	0.89 ± 0.2
MS- COCO	ALBEF	ACMR [34]	64.3 ± 0.7	0.062 ± 0.5	28.1 ± 0.6	0.65 ± 0.3	3.0 ± 0.0	0.57 ± 0.4
		SymUnimodal	59.7 ± 0.8	0.045 ± 0.4	30.1 ± 0.5	0.70 ± 0.2	2.8 ± 0.1	0.65 ± 0.3
		**Ours**	79.5 ± 0.7	0.045 ± 0.3	30.3 ± 0.4	0.81 ± 0.2	2.3 ± 0.1	0.87 ± 0.2
Flickr30K	FLAVA	Co-Attack [12]	61.3 ± 0.8	0.055 ± 0.4	29.2 ± 0.5	0.71 ± 0.3	3.0 ± 0.0	0.60 ± 0.4
		ACMR [34]	58.9 ± 0.9	0.060 ± 0.5	28.5 ± 0.6	0.63 ± 0.3	3.0 ± 0.0	0.55 ± 0.4
		**Ours**	78.9 ± 0.8	0.040 ± 0.3	31.8 ± 0.3	0.83 ± 0.2	2.2 ± 0.1	0.88 ± 0.2

**Table 2:** Ablation study (CLIP ViT-L/14 on MS-COCO). All variants use the full pipeline except the specified component.

Configuration	ASR (%)	SAS	LPIPS
Full Framework	82.3	0.89	0.038
w/o Symmetry Pairs (random *g*)	54.9	0.60	0.042
w/o Feature Hallucination (direct PGD)	62.5	0.71	0.053
w/o Adaptive Weighting (fixed λ,μ)	67.5	0.78	0.040
Geometric Symmetries Only	69.9	0.76	0.039
Syntactic Symmetries Only	68.0	0.75	0.039

**Table 3:** Correlation between vulnerability metrics and ASR. High SPR indicates the model over-relies on symmetry features.

Model	SAE	SPR	MRA	ASR (%)
CLIP ViT-L/14	0.32	0.68	0.25	82.3
ALBEF	0.29	0.65	0.22	79.5
FLAVA	0.27	0.62	0.20	78.9
SymTrain (Robust)	**0.18**	**0.72**	0.15	62.3
MMCert (Defense)	0.21	0.51	0.18	58.7

**Table 4:** Cross-Model Symmetry Transferability. Symmetry pairs discovered on one model effectively transfer to others, indicating shared reliance on symmetry priors.

Discovery Model	Target Model	ASR (%) ↑	SAS ↑	LPIPS ↓	BLEU-4 ↑
CLIP	CLIP	82.3	0.91	0.042	0.83
ALBEF	CLIP	76.8	0.87	0.045	0.82
CLIP	FLAVA	79.1	0.89	0.048	0.81
FLAVA	FLAVA	80.5	0.90	0.046	0.82

## Data Availability

Publicly available datasets were analyzed in this study. This data can be found here: MS-COCO (https://cocodataset.org, accessed on 1 April 2026) and Flickr30K (http://shannon.cs.illinois.edu/DenotationGraph/, accessed on 1 April 2026). The code presented in this study is available on request from the corresponding author.
